# Comment on “Living Donor Liver Transplantation With Augmented Venous Outflow and Splenectomy: A Promised Land for Small Left Lobe Grafts”

**DOI:** 10.1097/AS9.0000000000000243

**Published:** 2023-03-03

**Authors:** Kin Cheung Ng, Abdul R. Hakeem, Raj Prasad

**Affiliations:** From the Department of HPB and Transplant Service, St. James’s University Hospital, Leeds, United Kingdom.

We would like to congratulate the authors for publishing this important article on the outcome of utilizing left lobe grafts in their series of living donor liver transplantation (LDLT).^1^ Their study represents one of the largest series of utilizing left lobe grafts in the West and showed favorable 1-, 3-, and 5-year graft survival of 97%, 94%, and 89% comparable to the right lobe graft survival of 99%, 96%, and 93% (*P* = 0.97). This contrasts sharply from the European experience of 46 left lobe LDLTs from 4 centers showing a relatively poor 1-year graft survival of 59.4% due to a high rate of small for size syndrome (SFSS) and hepatic artery thrombosis.^2^ The key to their excellent outcomes was achieved by the use of venous outflow augmentation and liberal use of splenectomy to modulate portal inflow.

From their series, 61 of 130 (47%) recipients received left lobe graft. Donor/recipient selection likely played a huge role to their highly favorable outcomes. The Kyoto group previously showed that the overall survival was significantly worse when using left lobe graft compared with right lobe graft in recipients who were considered high risk (at least 2 risk factors of: model for end-stage liver disease [MELD] score > 20; preoperative hospitalization; recent preoperative bacterial infection).^3^ In contrast, no difference in survival were found between left lobe or right lobe recipient if only 1 risk factor was present. The Kyushu group reported donor age ≥ 48 years, MELD score ≥ 19, and end portal venous pressure ≥ 19 mm Hg as the important factors for the development of SFSS.^4^ These factors reflect on the poor graft quality from higher donor age, poor clinical status of the recipient and excessive portal flow after reperfusion of the partial graft. To highlight the important of graft selection, the Kyushu group has also devised a scoring system that incorporates factors such as graft size, donor age, MELD score, and the presence of portosystemic shunts, which correlate well with graft survival and can be employed for graft selection.^5^ Although there is currently no consensus on the selection criteria when comes to choosing left lobe graft, most groups have demonstrated that donor age, and MELD score as crucial deciding factors as mentioned. In fact, the European cohort with poor outcome with left lobe grafts reported MELD score >14 as the most significant factor for the development of SFSS.^2^ Looking at the Cleveland cohort, it is very clear that this is a highly selected group of donors and recipients when considering for left lobe graft with a donor median age of 36 years (interquartile range, 31–41 years), low median MELD score of 12 (interquartile range, 10, 16), and less severe portal hypertension as evident by the low median splenic volume (611 mL for left lobe graft compared with 918 for right lobe graft). Their left lobe graft experience has been accumulated over 8 years from 2013 to 2020 sharing between the 2 centers ranging from 5 to 17 left lobe grafts per year (mean 7 to 8 per year). This likely represents only a fraction of their overall liver transplant activity and further suggests that the recipients chosen for left lobe graft were highly selected.

As regards to the use of inflow modulation such as splenectomy in this series, a group from Tokyo has described in their series of 42 LDLTs using exclusively left lobe graft without the use of any inflow modulation technique with a very good 1-, 3-, and 5-year graft survival rate of 100%, 97%, and 91%, despite 38% of their recipients had graft-to-recipient weight ratio (GRWR) of less than 0.8%.^6^ Therefore, it is unclear how much contribution to current reported outcomes were related to patient selection or the use of splenectomy, the use of which has been shown to be a protective for graft dysfunction here. Although the reported morbidities with splenectomy were acceptable in this series, with sicker recipients, the added morbidity of splenectomy may become problematic and could lead to worse outcomes.

It has been well established that the grafts are considered small for size when the GRWR is less than 0.8% or the standard liver volume is less than 35%.^7^ This article has also reported the graft outcome when actual size of the graft was smaller than the expected minimum GRWR of 0.6%. While the 5-year graft survival of 88% and only 1 mortality amongst the 9 left lobe recipients is impressive, this is still small numbers, from selected donors and recipients. Until there are more data and replication of this practice in other centers, we feel that the usage of small left lobe grafts remains an option for very few and not a promised land for the many.
